# A Bioreactor for 3D *In Vitro* Modeling of the Mechanical Stimulation of Osteocytes

**DOI:** 10.3389/fbioe.2022.797542

**Published:** 2022-03-25

**Authors:** Koh Meng Aw Yong, Eric Horst, Dylan Neale, Sonya Royzenblat, Joerg Lahann, Colin Greineder, Megan Weivoda, Geeta Mehta, Evan T. Keller

**Affiliations:** ^1^ Department of Urology, Michigan Medicine, University of Michigan, Ann Arbor, MI, United States; ^2^ Department of Material Science and Engineering, University of Michigan, Ann Arbor, MI, United States; ^3^ Department of Chemical Engineering, University of Michigan, Ann Arbor, MI, United States; ^4^ Biosciences Institute, University of Michigan, Ann Arbor, MI, United States; ^5^ Department of Emergency Medicine, University of Michigan, Ann Arbor, MI, United States; ^6^ Department of Periodontics and Oral Medicine, University of Michigan, Ann Arbor, MI, United States

**Keywords:** prostate cancer, osteocyte, bioreactor 3D cell culture, mechanotrasduction, osteoblast (OB)

## Abstract

The bone is a mechanosensitive organ that is also a common metastatic site for prostate cancer. However, the mechanism by which the tumor interacts with the bone microenvironment to further promote disease progression remains to be fully understood. This is largely due to a lack of physiological yet user-friendly models that limit our ability to perform in-depth mechanistic studies. Here, we report a tunable bioreactor which facilitates the 3D culture of the osteocyte cell line, MLO-Y4, in a hydroxyapatite/tricalcium phosphate (HA/TCP) scaffold under constant fluidic shear stress and tunable hydrostatic pressure within physiological parameters. Increasing hydrostatic pressure was sufficient to induce a change in the expression of several bone remodeling genes such as Dmp1, Rankl, and Runx2. Furthermore, increased hydrostatic pressure induced the osteocytes to promote the differentiation of the murine macrophage cell line RAW264.7 toward osteoclast-like cells. These results demonstrate that the bioreactor recapitulates the mechanotransduction response of osteocytes to pressure including the measurement of their functional ability in a 3D environment. In conclusion, the bioreactor would be useful for exploring the mechanisms of osteocytes in bone health and disease.

## Introduction

The bone is a mechanosensitive organ commonly colonized by tumor cells in metastatic cancer (Bubendorf et al., 2000). In advanced prostate cancer, the majority of bone lesions present as osteoblastic; however, there is frequent underlying osteoclastic activity (Logothetis and Lin, 2005; Sottnik and Keller, 2013). Understanding how metastatic prostate cancer lesions contribute to pathological bone remodeling is not fully understood, in part, due to the lack of *in vitro* model systems that are easily manageable and physiologically relevant.

Multiple forces are exerted on the bone under healthy or diseased conditions, and these forces include pressure and shear stress (Stevens et al., 2006; Lau et al., 2010; Yavropoulou and Yovos, 2016; Stewart et al., 2020). Separate studies have shown that increasing pressure can drive the differentiation of mesenchymal stem cells (MSCs) toward osteogenic lineage and stimulate bone cells to increase the expression of osteopontin and decrease osteoprotegerin (OPG), an inhibitor of osteoclasts (Klein-Nulend et al., 1995). The application of fluid shear stress (FSS) has been demonstrated to increase alkaline phosphatase and mineralization activity in progenitor cells such as MSCs (Datta et al., 2006; Delaine-Smith et al., 2012). FSS on osteocytes induces the secretion of cytokines and growth factors that promote further osteogenic differentiation in MSCs (Hoey et al., 2011), although there is also a suggestion that FSS can induce bone remodeling independent of osteocytes (Kwon et al., 2012). In prostate cancer, it has been shown that tumors growing in mouse tibiae increase intraosseous pressure (Sottnik et al., 2015). This increase in pressure induced osteocytes to increase CCL5 expression that promoted tumor expansion. Taken together, these findings highlight the importance of incorporating mechanical forces in any bone models developed for studying the bone under healthy or diseased conditions.

In this article, we describe a method to construct a tunable bioreactor using materials readily available in most biological research laboratories. This system allows the inclusion of fluid flow and the ability to modulate pressure. To further mimic *in vivo* growth conditions, MLO-Y4 osteocyte-like cells and MC3T3 osteoblast-like cells were cocultured with beads made of hydroxyapatite/tricalcium phosphate (HA/TCP), a major component of the mineralized bone. We further show that systematically modulating pressure in this bioreactor under constant FSS can induce these cells to secrete factors that promote osteoclastogenesis in RAW264.7 cells. This demonstrates the utility of the bioreactor platform as a versatile research tool in studying the interplay among mechanical forces in driving bone development processes.

## Materials and Methods

### Cell Culture

MLO-Y4 osteocyte-like cells (Kato et al., 1997) (kindly provided by Dr. Lynda Bonewald, Indiana University) were cultured in α-DMEM (Gibco, Thermo Fisher, Waltham, MA, United States) supplemented with 5% fetal bovine serum and 5% bovine calf serum with 1% penicillin/streptomycin. Growth media was changed every three days, and cells were passaged upon reaching 80% confluence where the cells were seeded on a new 100 mm dish at a 1:10 dilution. MC3T3-E1 cells (American Type Culture Collection (ATCC), Manassas, VA, United States) were cultured in DMEM (Gibco, Thermo Fisher) supplemented with 10% FBS and 1% penicillin/streptomycin. Growth media was changed every three days, and cells were passaged every 7–10 days and seeded on a new 100 mm dish at a 1:10 dilution. Both cell lines were grown under 37°C and 5% CO2. RAW264.7 cells (ATCC) were cultured in DMEM (Gibco, Thermo Fisher) supplemented with 10% FBS and 1% penicillin/streptomycin. Growth media was changed every three days, and cells were passaged upon reaching confluency to a fresh 100 mm dish at a 1:10 dilution. Cell identities were confirmed every 6 months using short tandem repeat (STR) analysis.

### Bioreactor Fabrication

The bioreactor consists of two cylindrical polydimethylsiloxane (PDMS) chambers (upper and lower) separated by a 0.2 mm thick and 1 µm pore size Omnipore filter membrane (Millipore, Sigma Aldrich, Burlington, MA). The ratio of PDMS to crosslinker ratio used to fabricate all molds and the device was 10:1. To make the mold for constructing the upper chamber, a single well of a 48-well plate was first filled with unpolymerized PDMS. To make the mold for the lower chamber, a single well of a 96-well plate was also filled with unpolymerized PDMS and left to cure at 60°C oven overnight. The mold was removed from the well by inserting a fine-tipped weighing spoon that was wet in ethanol between the wall of the well and cured PDMS. The weighing spoon was used to dislodge the mold from the well by moving it around the wall. After removing the mold, 10 mm of PDMS from the top of the 24-well PDMS mold was removed with a clean razor and 5 mm of PDMS from the top of the 96-well PDMS mold was removed. To fabricate the upper chamber, two 0.2 mm wide needles of 30 mm each in length were inserted into the cut side of 48-well PDMS so that the needles stand perpendicular to the cut surface at 5 mm apart. The 48-well PDMS/needle was placed into a single well of a 12-well plate with the needles facing upward. To fabricate the lower chamber, a single 0.2 mm wide needle of 30 mm length was inserted into the cut side of the 96-well PDMS so that the needle stands perpendicular to the cut surface. The 96-well PDMS/needle was then placed into a single well of a 48-well plate with the needle facing upward. Unpolymerized PDMS was used to fill the rest of the chambers and left to cure in a 60°C oven overnight. The needles were first removed from the cured PDMS before removing the PDMS block from the wells the same way as described earlier. To separate the PDMS mold from the chamber, a sharp tool was used to generate a small separation at the interface between the mold and chamber at the bottom. The interface was visible under good lighting. The wall of the chamber was gently teased apart from the mold. Next, a fine-tipped weighing spoon wet in ethanol was used to remove the mold by moving the spoon along the inner wall of the chamber. Push firmly on top of the chamber to push out the mold. The same procedure was applied to separate the 96-well PDMS mold for the lower chamber. The resultant upper chamber was a cylindrical PDMS block with a cylindrical cavity with two open channels running vertically into the top wall of the block. The resulting bottom chamber was a cylindrical block with a cylindrical cavity and a single-open channel running vertically into the top wall of the block (Supplementary Figure S1).

To assemble the upper and lower chambers into the functional bioreactor, a single piece of 0.2 mm thick filter membrane (Omnipore, Millipore) was placed between the open cavities of both the upper and lower chambers. The filter was fixed in place by squeezing the lower chamber into the cavity of the upper chamber, and an excess filter membrane was trimmed with a sharp blade (Supplementary Figure S1). The upper and lower chambers were sealed by applying unpolymerized PDMS between the interfaces followed by curing at 60°C overnight. The finished bioreactor was sterilized using an autoclave at 121°C and 15 psi.

### Reservoir Fabrication

To fabricate the reservoir, two needles of 200 µm in diameter were inserted into a 0.5 mm thick piece of round PDMS mold with the diameter of a single well in a 48-well plate. The needles were placed such that they run perpendicular to the flat surface of the PDMS mold, and the PDMS/needles were placed into a single well of a 24-well plate. Unpolymerized PDMS was poured to fill the well, covered the PDMS mold, and left to cure at 60°C overnight.

The cured 24-well PDMS was removed using the fine-tipped weighing spoon as described earlier. The needles were removed, and the 0.5 mm thick 48-well PDMS mold was removed using the weighing spoon. The top half of the 24-well PDMS block was removed using a sharp blade. In total, two pieces of 150 mm long PTFE tubing (Cole-Palmer, Vernon Hills, IL, United States) were inserted into each of the channels created by the needles in the 24-well PDMS cylindrical block, and one of the tubings was threaded through one of the two channels such that a 30 mm length of tubing extends from the bottom end of the 24-well block. The other tubing was threaded through the second channel such that the end does not extend past the bottom opening (Supplementary Figure S1). The 24-well PDMS block with tubing was plugged into a capless 5 ml microfuge tube (Eppendorf) and sealed using PDMS. The reservoir was then heat sterilized at 121°C and 15 psi.

### Size Sorting of Hydroxyapatite/β-Tricalcium Phosphate Beads

The stock HA/TCP beads (CaP Biomaterials, East Troy, WI, United States) consisted of beads with diameters ranging from 10 to 97 µm. As the interstitial space between osteocytes in the bone is 25 µm [ref], 25 µm beads were enriched for using two sieves of pore sizes 25 and 20 µm (Thomas Scientific) (Supplementary Figure S2). A total of 1 g of unsorted beads was first suspended in 50 ml of 95% ethanol. The suspended beads were first passed through the 25 µm sieve to remove beads with diameters above 25 µm. The flow through was next passed through the 20 µm sieve to remove beads with diameters below 20 µm. The retained beads on the 20 µm sieve were collected and suspended in 75% ethanol for sterilization. The ethanol was replaced with sterile water by first pelleting the beads using centrifugation (300 g at 5 min). The supernatant was aspirated away, and the pellet was resuspended in sterile water. This process was repeated three times to remove residual ethanol. A small aliquot of beads was used for determining the bead density and size using light microscopy (EVOS, Life Technologies) (Supplementary Figure S2).

### Seeding the Bioreactor With Cells and HA/TCP Beads

**FIGURE 1:**
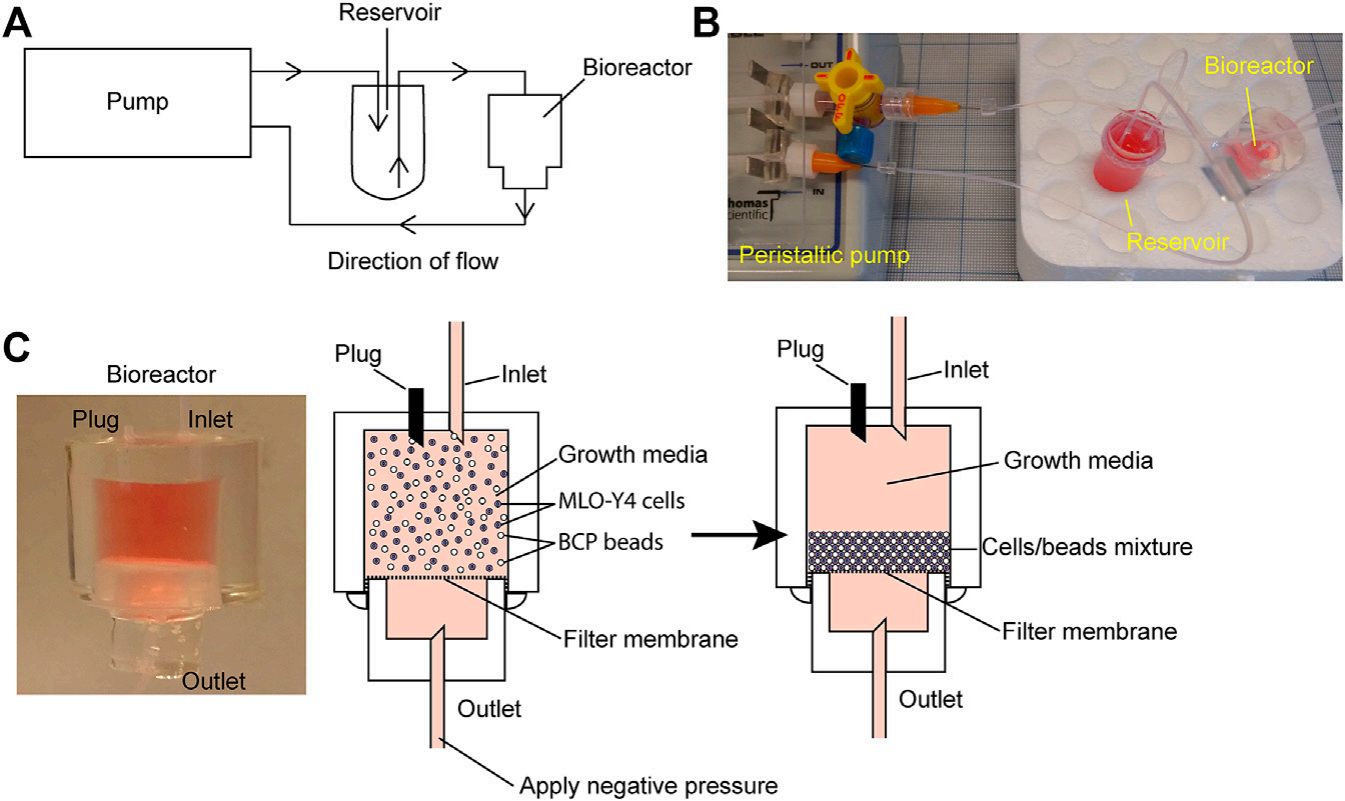
Bioreactor working model. **(A)** Schematic of the bioreactor system set up. **(B)** Image of the bioreactor set up with a four-way stopcock introduced into the circuit as a method of increasing system hydrostatic pressure. **(C)** Close-up image of the working bioreactor module (left panel); beads/cells suspension before packing (middle panel); and beads/cells suspension after packing (right panel).

First, 1 × 10^7^ HA/TCP beads were pre-treated with 750 µg rat tail collagen I (Corning, Corning, NY) in 5 ml of 2 mM acetic acid at 4°C for 1 h on an orbital shaker. Before adding cells, the beads were pelleted at 300 g for 5 min before resuspending in 10 ml of a fresh growth media to neutralize pH levels. MC3T3 or MLO-Y4 cells were then mixed with HA/TCP beads in a 1:5 cell:bead ratio. The cell/bead suspension was compacted using centrifugation at 300 g for 5 min. The supernatant was aspirated away carefully without disturbing the pellet after which the pellet was resuspended in a fresh growth media to generate a cell/bead suspension with a density of 4 × 10^6^ cells/ml and 2 × 10^7^ beads/ml. Before seeding the bioreactor, the membrane separating the upper and lower chambers of the bioreactor was first wetted using the growth media. The upper chamber was filled with 500 µL of media and negative pressure was applied at the bottom chamber using a syringe with a dispensing needle (23G) attached to 24G PTFE tubing (Cole-Palmer) to draw the growth media through the membrane. Negative pressure was applied until the upper chamber was empty of the growth media. Next, 500 µL of the cell/bead suspension was applied to the upper chamber using a syringe and blunt tip dispensing needle (23G) though one of the open channels in the upper chamber and negative pressure was applied at the bottom chamber to compact the suspension into a pellet that forms on the filter membrane. After forming the pellet, the upper chamber was again filled with the fresh growth media. Care was taken during this step, that is, not to disturb the formed pellet at the membrane. Once the upper chamber was filled with media, one of the open channels at the upper chamber became closed with a 10 mm long occluded 24G PFTE tubing (Figure 1C).

For negative TC control, 1 × 106 cells and 5 × 106 beads were plated on a 100 mm TC plate. Cells were allowed to attach and grow under 37°C, 5% CO_2_ conditions until harvest.

### Setting up the Bioreactor to the Peristaltic Pump and Reservoir

The peristaltic pump tubing was connected to a 1.5 mm male luer lock (Cole-Palmer) with an attached dispensing needle (23G). A 100 mm long PTFE tubing (24G) was attached to the tip of each dispensing needle. The tubing was next flushed with the growth media to remove air bubbles.

The reservoir was filled with the growth media using the PTFE tubing that extends into the 5 ml Eppendorf tube using a 10 ml syringe/blunt tip dispensing needle (23G). Once the reservoir was filled, the syringe/dispensing needle was removed and the same tubing was inserted into the second channel opening in the bioreactor upper chamber. The second tubing in the reservoir was attached to the PTFE tubing on the dispensing needle (23 G) attached to the peristaltic pump (Fisherbrand Variable-Flow Peristaltic Pump, Catalog No. 13-876-4; FisherScientific, Waltham, MA, United States) facing the outward flow direction using an adapter consisting of a 20 mm long PDMS block with a 200 µm channel. The remaining 100 mm long PTFE tubing on the dispensing needle (23G) attached to the peristaltic pump facing the inward flow direction was connected to the bottom chamber of the bioreactor to complete the circuit (Figures 1A,B). Once the circuit has been completed, the peristaltic pump is turned on to provide a fluid flow of 30 μL/min.

### Modulating and Measuring Internal Hydrostatic Pressure

**FIGURE 2:**
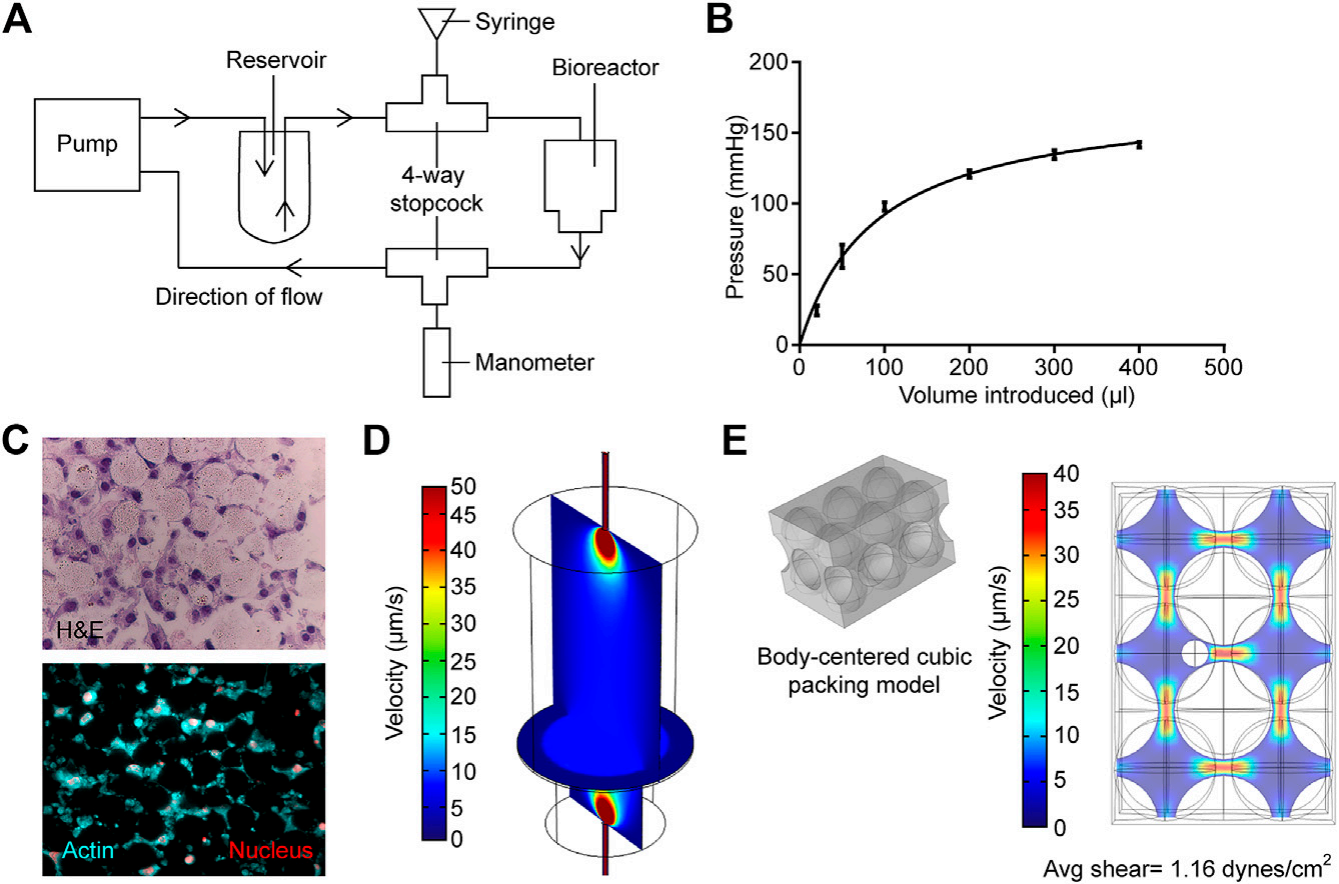
Measurement and modeling of system pressure and fluid shear stress. **(A)** Schematic of how system is modified to measure and modulate internal pressure. **(B)** Measured internal pressure in response to the additional fluid volume introduced. **(C)** H&E sections (upper panel) and actin staining (bottom panel) of the MLO-Y4 murine osteocyte-like cell/bead disc structure **(D)** Rendered vertical and horizontal slices showing medium flow profiles within the primary bioreactor model. **(E)** Left; profile of hard-packed HA/TCP spheres. Right; rendering of the medium velocity profile around the modeled osteocyte, larger spheres are HA/TCP beads.

To modulate the hydrostatic pressure within the bioreactor, a four-way stopcock (Smiths Medical, Minneapolis, MN, United States) was introduced into the fluid flow line just at the outward flow line at the peristaltic pump (Figure 2A). The stopcock serves as the inlet for introducing additional fluid volume *via* a 1 ml syringe.

To measure the change in pressure with additional fluid volume, another four-way stopcock was introduced into the fluid flow line at the inward flow line at the peristaltic pump (Figure 2A). This stopcock was attached to an intra-compartmental pressure monitor (Stryker) that was modified to fit into the stopcock. Before measuring the pressure, the manometer was first placed on a level surface and calibrated. Additional fluid volume was added through the inlet at the outflow line, and the change in pressure was recorded after 1 min once the reading has stabilized. Fluctuations in pressure were not observed during peristaltic pump activity. Furthermore, there was no leakage from the devices at the pressures used.

### Modeling Fluid Shear Stress

Computational simulations were performed with the commercially available finite element package, COMSOL (v 5.2a, Burlington MA), to verify physiological levels of shear stress within the bioreactor. Free and porous media flow and laminar flow physics modules were utilized in each model respectively, as described previously (Novak et al., 2019).

The bioreactor medium flow was simulated in the primary model (Figure 2D), calculating the average fluid velocity entering the porous HA layer. A fluid velocity of 2.55 mm/s and a pressure of 0 pa were used as input and output for the primary model, respectively. A mercury porosimeter (MicroActive AutoPore V9600 Version 1.02) was utilized to measure the permeability of the HA layer; briefly, the HA powder was pressed, lyophilized, and analyzed for the pore structure. A 5 cm^3^ stem volume was utilized at a mercury temperature of 18.93°C for porosimetry. The permeability and porosity of 1.97 × 10–12 m^2^ and 0.2075 were measured and input into the model, respectively.

### Quantitative Polymerase Chain Reaction

Total cell RNA was isolated using RNeasy (Qiagen, Hilden, Germany) and complementary DNA (cDNA) was synthesized using the iScript cDNA synthesis kit (Bio-Rad, Hercules, CA, United States) using 1 µg of total RNA. Quantitative polymerase chain reaction was performed on Applied Biosystems QuantStudio 5 (Thermo Fisher) using the Power SYBR Green Master Mix (Applied Biosystems, Thermo Fisher). All primers were obtained from Qiagen and pre-validated by the manufacturer. The following bone remodeling genes of mouse origin were analyzed: AlpI (Qiagen), Dmp1 (Qiagen), Rankl (Qiagen), Runx2 (Qiagen), and Sost (Qiagen). GAPDH (Qiagen) or β-actin (Qiagen) was used as reference genes. To evaluate for changes in GAPDH and β-actin mRNA expression in MLO-Y4 cells exposed to pressure, 1 × 10^5^ cells were plated into 2 ml of complete media in 6-well cell culture plates. After 24 h of incubation, the cells were subjected to constant 0, 20 mmHg or 40 mmHg for 72 h using the FX-5000C Compression System (Flexcell International Corporation, Burlington, NC, United States), at which time total RNA was collected and 1 µg of total RNA was used to create cDNA and subjected to PCR for GAPDH and β-actin mRNA expression as described earlier. Samples were run in triplicate. Data are reported of as mean ± SD threshold cycle (Ct).

### Immunoblot

To harvest the pellet, the bioreactor was first dismantled using a clean dissecting blade to make two vertical cuts along the upper chamber wall. The upper chamber was peeled apart, and the cells/bead pellets were removed intact from the filter membrane as a disc using a beveled cell lifter.

To isolate protein lysate, the cell/bead pellet was treated with RIPA buffer (Thermo Fisher) supplemented with 1 × HALT protease and phosphatase inhibitors (Thermo Fisher). The cell lifter was used to first break apart the pellet followed by the application of ultrasound sonication to further disrupt the lysate and increase protein yield.

To harvest the negative TC control, growth media was first aspirated away followed by two washes with 1 × PBS. The final PBS wash was aspirated away, and 500 µL of RIPA buffer supplemented with 1 × HALT protease and phosphatase inhibitors (Thermo Fisher) was added to the TC plate. A cell lifter was used to scrape the cells and beads off, and the 100 mm TC plate followed by the application of ultrasound sonication to disrupt the cell lysate and improve protein yield.

Before starting the immunoblot, the cell lysates were first centrifuged at 1,500 g for 10 min to pellet the HA/TCP beads. The protein lysate was then mixed with 2 × Laemmli-loading buffer (Bio-Rad) and subjected to electrophoresis on a 4–15% gradient gel (Bio-Rad) followed by a transfer to the nitrocellulose membrane using the Trans-Blot Turbo system (Bio-Rad). After transfer, the membrane was blocked with 5% milk in 1 x tris-buffered saline (TBS) with 0.05% triton X-100 at 4°C overnight. Primary antibody dilutions are as follows: sheep antimouse Dmp1 (R&D Minneapolis, MN, United States) are diluted 1:500; goat antimouse Rankl (BioVision, Milpitas, CA, United States) were used at a 1:1,000 dilution; rabbit antimouse Runx2 (Cell Signaling, Danvers, MA, United States) were used at a 1:1,000 dilution; and rabbit antimouse actin (Cell Signaling) were used at a 1:1,000 dilution.

### Harvesting Conditioned Media From the Bioreactor Reservoir

Conditioned media (5 ml) was harvested aseptically from the reservoir by peeling apart the PDMS cap on the 5 ml microfuge tube and aspirating the media from the tube into an Amicon® Ultra-15 centrifugal filter unit 100 kDa (Millipore Sigma). The conditioned media was concentrated at 4,000 g for 5 min to a final volume of 500 µL, which allows for the addition of a small volume of conditioned media during experiments so that we can use fresh media and supplements.

### Treatment of RAW264.7 Cells With Conditioned Media From the Bioreactor

RAW264.7 cells were first plated onto a 24-well plate at a density of 1.5 × 10^5^ cells/cm^2^ in a final volume of 0.5 ml of DMEM supplemented with 10% FBS and 1% penicillin/streptomycin and no additional factors. Then, 50 µL of conditioned media from each condition was added to the growth medium immediately followed by the plating of cells, and cells were incubated for 3 days at which time they were stained for tartrate-resistant acid phosphatase (TRAP).

### Tartrate-Resistant Acid Phosphatase Staining

TRAP staining was performed according to the manufacturer’s instructions using the Acid Phosphatase, Leukocyte (TRAP) Kit (Millipore Sigma). First, growth media-containing conditioned media was aspirated away from the wells, and the cells were gently washed twice with 1 × phosphate-buffered saline (PBS) prewarmed to 37 C. Next, the cells were fixed with fixative solution (25% sodium citrate, 65% acetone, and 3% formaldehyde) for 30 s before removing fixative followed by two washes with deionized water prewarmed to 37 C. Next, the cells were stained with the staining solution (containing diazotized Fast Garnet GBC solution, naphthol AS-BI phosphate solution, and acetate and tartrate solution) for 1 h in a 37 C incubator. After 1 h, the staining solution was removed, and cells were rinsed with deionized water followed by a hematoxylin counterstain for 30 s. The hematoxylin counterstain was removed, and counterstained cells were rinsed with deionized water. The stained cells were first air dried and images were acquired using light microscopy (EVOS, Life Technologies).

### Statistical Analysis

All experiments were repeated using three independent bioreactors. Data are reported as mean ± standard deviation (SD). Statistical analysis was performed using Prism 5 (GraphPad Software, La Jolla, CA). Multiple groups were compared using one-way ANOVA with Bonferroni post-test. Data comprising only two groups were analyzed using Student’s *t*-test. For all statistical analyses, *p* ≤ 0.05 was determined as statistically significant.

## Results and Discussion

### Physiological Levels of Shear Stress and Pressure Can Be Maintained in the Bioreactor

The bioreactor in the current study was based on previous studies, in which an osteoblast-like cell line or primary cells derived from bone chips were cultured in 3D on packed hyaluronic acid beads under slow perfusion (Gu et al., 2015; Sun et al., 2015; Sun et al., 2017; Choudhary et al., 2018). In the current study, the ability to modulate pressure and evaluate the impact of pressure on osteocytes’ activity provided a novel method to better recapitulate the physiological state of the bone. Previous measurements of pressure within the mouse tibiae demonstrated that levels vary between 20 mmHg at basal levels up to 40 mmHg when tumor is growing within the marrow (Sottnik et al., 2015). To determine if the bioreactor could replicate these physiological levels, we measured how hydrostatic pressure in the bioreactor changed with increasing fluid volume. The sealed system was sensitive to small changes in fluid volume, and the introduction of 25 µL of additional fluid volume was sufficient to induce an increase in pressure by 25 mmHg. A rapid increase in pressure was observed up to 100 µL of added fluid, but the rate of increase diminished with additional volume (Figure 2B). This was likely due to the upper limit of the intra-compartmental pressure monitor. These data demonstrated that the bioreactor could provide physiological levels of pressure.

### MLO-Y4 Cells Embedded in the HA/TCP Scaffold Maintain Cell–Cell Contact Under Bioreactor Growth

The use of HA/TCP beads provided a 3D growth scaffold and a bone-like environment for cell culture. We first determined the cellular organization of osteocyte MLO-Y4 cells embedded within the HA/TCP scaffold. Cells were cultured for 3 days under FSS with no additional pressure. The bioreactor was disassembled after 3 days of culture, and the cell/scaffold pellet was isolated. The pellet containing cells and beads was readily isolated intact within 3 days of culture. It was then formalin fixed and processed into histological slides. H&E staining of the sections indicated that the HA/TCP beads were arranged in a matrix with the MLO-Y4 cells embedded within the space between the beads. Staining also indicated that neighboring cells contacted each other (Figure 2C), while staining for actin cytoskeleton using fluorescent phalloidin did not yield stress fibers, the staining pattern indicated the presence of extensive cell–cell interactions (Figure 2C). These results demonstrated that similar to osteocytes within the bone, the osteocytes within the bioreactor had cell-to-cell contact; however, but it is unclear if they form a network similar to osteocytes within the bone.

To ensure the physiologically relevant levels of FSS recreated within the bioreactor, we used computational modeling. Briefly, the fluid velocity, calculated from the first model, was then used as input for the sequential model (Figures 2D,E) to allow the estimation of the maximum shear stress experienced by osteocytes within the HA hard-packed spheres. Hematoxylin and eosin cross sections of the HA spheres and osteocytes were prepared and stained to determine the average diameters of both the spheres (25 µm) and osteocyte cells (7 µm) (Figure 2C). A common hard sphere packing formation, body centered cubic (BCC), was used to model the HA layer. A sphere representation of an osteocyte cell was then modeled into the largest of the BCC interstitial sites (Figure 2E). A maximum shear stress of 2.44 dynes/cm^2^ was calculated. Finally, a mesh analysis was performed to ensure that the results of each model were independent of the element size (Supplementary Figure S3).

These models have several limitations, making multiple assumptions. The primary model shown in Figure 2D takes into account the measurable fluid velocities at both the inlet and outlet tubes, the dimensions of the bioreactor, and the porosity and permeability of the densely packed sphere region. When compiled, these values and geometries provide a gross estimation for the fluid profiles within the bioreactor. The porosity/permeability measurements of the compressed HA spheres were performed with a mercury porosimeter (MicroActive AutoPore V9600 Version 1.02; sample size = 5 cubic cm). Therefore, porosity and permeability were experimentally measured to inform Brinkman’s equations for free and porous media flow. However, we assumed that the sphere packing distribution is conserved within both the bioreactor and the mercury porosimeter, and by extension, also within the computational simulation, *in silico*. Furthermore, neither the cellular nor secreted ECM constituents were included in this primary simulation, as they had not been included in the samples measured *via* porosimeter (Figure 2D). We, therefore, assumed that cellular plus ECM components of the HA region do not pose a significant impact on the fluid flow profiles within the bioreactor, and measuring these values accurately using the methodology outlined before would pose a significant technical challenge. We, therefore, made an assumption that the permeability and porosity of the densely packed HA sphere region are approximately the same with or without the inclusion of osteocytes. In terms of the secondary model (Figure 2E), we also used a gross estimation, displaying a singular section of the cell-laden HA layer. Briefly, average velocity is calculated directly before the HA layer within the primary model. This velocity is then used as an input parameter for the secondary model (Figure 2D). Sphere packing configuration was assumed to be BCC, as it is one of the most common packing structures for similar-sized spheres. As observed in Figure 2C, HA spheres will not always form ordered configurations in 3D, and therefore the BCC is not always a perfect representation of the packing structure. However, we use BCC here as an estimation to sample a highly likely microscopic region of the overall HA layer. We would also like to highlight that the impact of all possible HA sphere configurations has already been attributed to the permeability and porosity measurements that inform the primary model (5 cm^3^ of compressed HA spheres measured *via* mercury porosimeter). Therefore, our second model input parameters are based on this assumption. It is likely for the regions of HA spheres to also fall within simple cubic (SC), hexagonal closed packing (HCP), and face-centered cubic (FCC) structures as well. However, due to the size of the osteocyte cells, SC and BCC are the structures to form most likely. This secondary model is used to inform an upper range of shear stresses experienced by cells within the bioreactor, while also ensuring the bioreactor produces a physiologically relevant range of fluid shear stress.

### Culturing MLO-Y4 or MC3T3 Cells in the Bioreactor Alone Affected Bone Remodeling Genes Dmp1, Rankl, and Runx2 Expression

**FIGURE 3:**
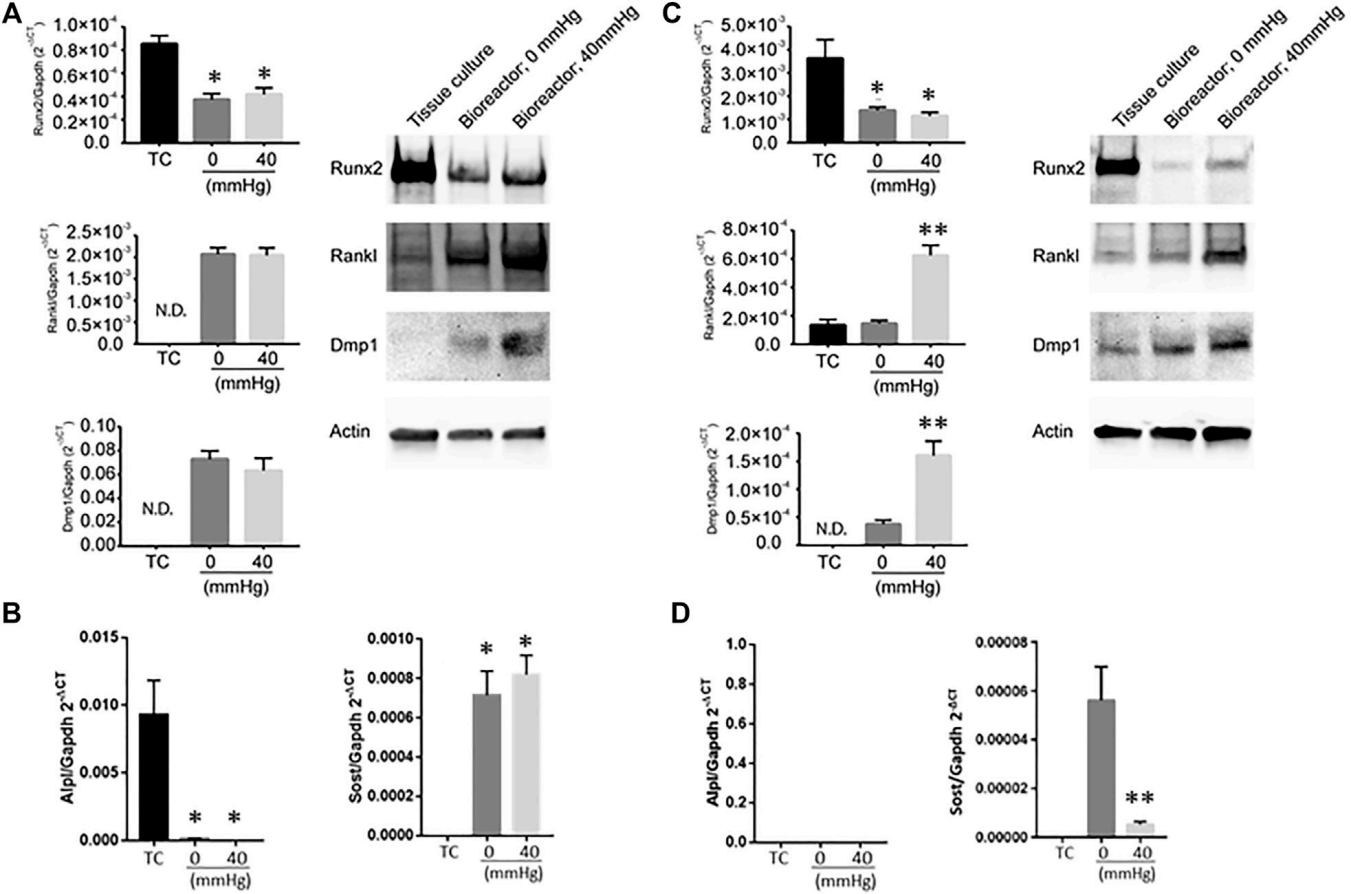
Expression of bone remodeling genes in response to changes in pressure. **(A)** Dmp1, Rankl and Runx2 mRNA levels (left) and protein (right) in MC3T3 when grown under different conditions (tissue culture plate (TC); bioreactor at 0 mmHg; bioreactor at 40 mmHg). **(B)** Alpl (left) and Sost (right) transcript levels in MC3T3 cells grown on TC, bioreactor with no pressure added (0 mmHg) or with 40 mmHg pressure added. **(C)** Dmp1, Rankl and Runx2 mRNA levels (left) and protein (right) in MLO-Y4 ′when grown under different conditions (TC; bioreactor at 0 mmHg; bioreactor at 40 mmHg). **(D)** Alpl (left) and Sost (right) transcript levels in MLO-Y4 cells grown on TC, bioreactor with no pressure added (0 mmHg) or with 40 mmHg pressure added. Data are presented as mean ± SD from three independent experiments. *p ≤ 0.05 vs. TC; **p < 0.05 vs. bioreactor at 0 mmHg.

The impact of hydrostatic pressure on osteocyte communication toward osteoblasts and osteoclasts has been previously described in murine models (Stevens et al., 2006; Lau et al., 2010; Kwon et al., 2012). Hind limb suspension or femoral vein ligation in a murine model to decrease or increase femoral intramedullary pressure, respectively, demonstrated that the alteration of pressure did not alter the oxygenation of osteocytes, and thus did not account for alteration in bone remodeling (Stevens et al., 2006), suggesting other mechanisms play a role on osteocyte-mediated bone remodeling. *In vitro* studies have provided clues as to what some of these factors may be. For example, hydrostatic pressure has been shown to induce prostaglandin production from chicken calvariae osteocytes (Kleinnulend et al., 1995). Additionally, cyclic hydrostatic pressure increased the intracellular concentration COX-2 and RANKL/OPG ration of MLO-Y4 cells (Liu et al., 2010). Taken together, these studies demonstrate that hydrostatic pressure alters osteocyte function. The ability to modulate hydrostatic pressure within the bioreactor enabled us to examine how variations in system pressure would affect bone cell phenotype. To determine if pressure-induced changes in the housekeeping genes, we subjected MLO-Y4 cells to 0, 20, or 40 mmHg for 72 h, then measured GAPDH and β-actin mRNA expression using quantitative PCR. Pressure for this duration did not impact the expression of the housekeeping genes (Supplementary Table S1), indicating they could be used for the normalization of mRNA expression. MLO-Y4 or MC3T3 cells were cultured in standard tissue culture plates or the bioreactor for 3 days with either 0 mmHg or 40 mmHg pressure. Three-dimensional coculture of osteoblast-like MC3T3 cells with HA/TCP beads within the bioreactor without additional pressure (0 mmHg) was sufficient to induce a change in phenotype characterized by the downregulation of Runx2 and the upregulation of Rankl and Dmp1 at both mRNA and protein levels compared to cells grown on a 2D tissue culture plate (Figure 3A). Downregulation of Alpl, a marker of osteoblasts was also observed at the transcript level while Sost, a marker of osteocytes, was upregulated in the bioreactor compared to standard tissue culture (TC) conditions (Figure 3B). Increasing the bioreactor’s system pressure to 40 mmHg did not further affect the expression of any of these factors (Figures 3A,B). We examined a range of pressure setting (0, 20, 40, and 60 mmHg) and found no significant changes to Runx2; whereas Rankl mRNA expression was increased by pressure and Dmp1 mRNA expression was decreased (Supplementary Figure S4). This was not reflected in the protein levels, which demonstrated no change, except a decrease in RANKL at 60 mmHg.

In MLO-Y4 cells, Runx2 was downregulated when cocultured with HA/TCP beads in the bioreactor without additional pressure (0 mmHg). Dmp1 was upregulated while no change in Rankl expression was observed (Figure 3C). No detectable Alpl transcript was observed while Sost transcript levels were detectable compared to TC conditions (Figure 3D). Increasing the pressure in the bioreactor to 40 mmHg induced a significant increase in Rankl expression and further increased the expression of Dmp1 (Figure 3C). Interestingly, at 40 mmHg pressure, Sost transcript levels decreased compared to 0 mmHg conditions (Figure 3D). When MLO-Y4 cells were subjected at a range of different pressure conditions (0, 20, 40, and 60 mmHg), an increase in Rankl expression was observed at 20 mmHg with no further changes at higher pressures. However, Dmp1 expression in MLO-Y4 was highest at 20 mmHg and decreased under 40 and 60 mmHg conditions (Supplementary Figure S4).

### Conditioned Media Harvested From Both Cell Lines Had the Ability to Induce the Formation of TRAP Positive Multinucleated Osteoclast-Like Cells From RAW264.7 Cells

**FIGURE 4:**
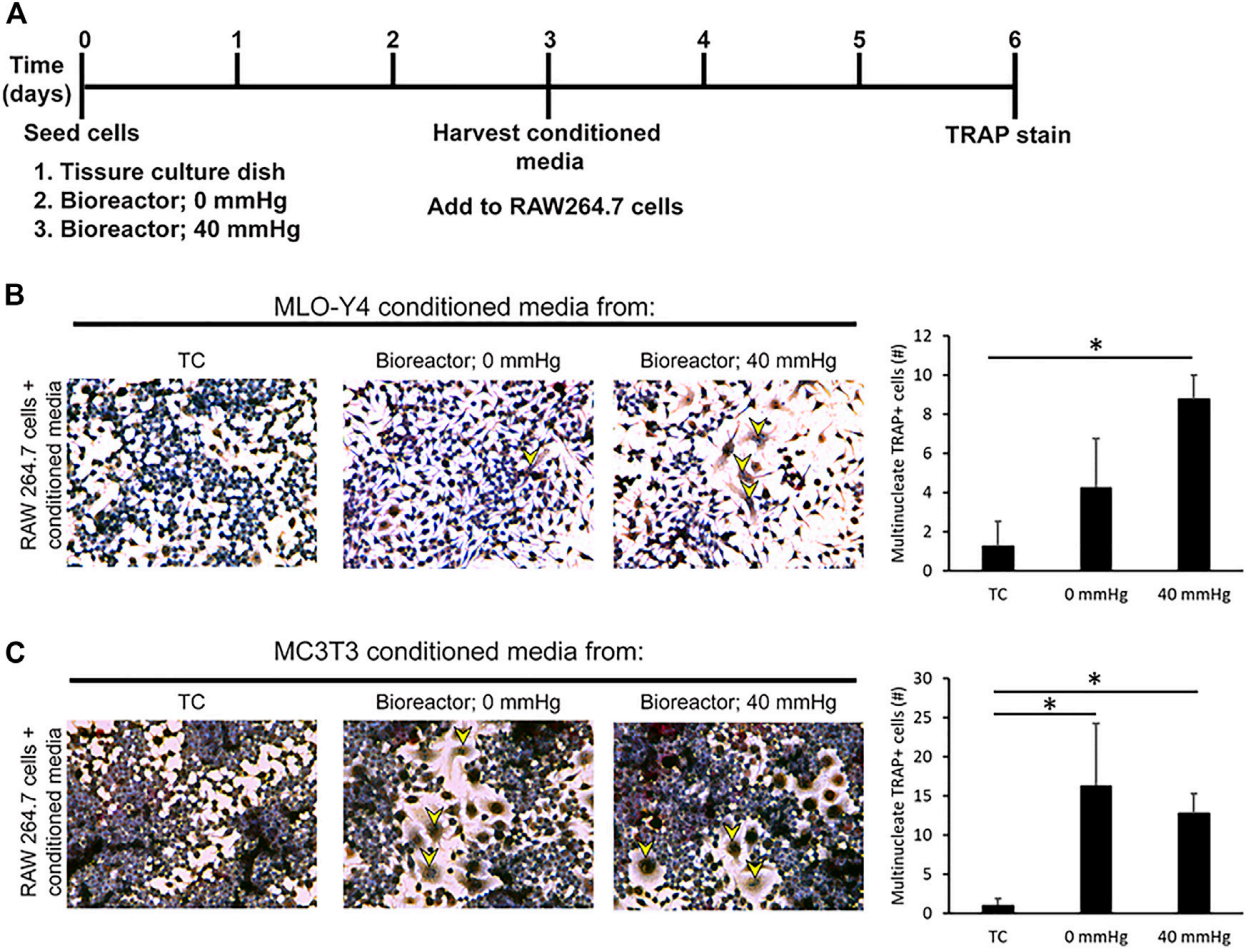
Conditioned media from bone cells grown in bioreactor induces the formation of multinucleated TRAP + cells. **(A)** Experimental set up. **(B)** Response and quantification of RAW264.7 cells incubated with an addition of MLO-Y4 conditioned media harvested from TC, bioreactor (0 mmHg), and bioreactor (40 mmHg). TRAP positive cells are indicated by yellow arrowheads. The number of TRAP + cells with 2 or more nuclei was counted. **(C)** Response of RAW264.7 cells incubated with an addition of MC3T3 conditioned media harvested from TC, bioreactor (0 mmHg), and bioreactor (40 mmHg). Data are presented as mean ± SD from three independent experiments. *p ≤ 0.05 vs. TC.

The decrease in Runx2 expression combined with an increase in Rankl and Dmp1 under pressurized conditions in MC3T3 and MLO-Y4 cell lines suggested that the cells shift toward a more osteocyte-like phenotype. To examine if this shift toward a more osteocyte-like phenotype affects biological functions such as osteoclastogenesis, conditioned media was harvested from MC3T3 and MLO-Y4 cells grown under three different conditions: TC, bioreactor (0 mmHg), and bioreactor (40 mmHg) (Figure 4A). The conditioned media was used to induce osteoclastogenesis in RAW264.7 cells, which are known to possess the ability to differentiate into multinucleated TRAP positive osteoclasts on TC plastic. Conditioned media from MLO-Y4 cells grown in the bioreactor was able to increase the percentage of multinucleated TRAP positive cells (1.3%) formed after three days compared to conditioned media from cells grown on TC plastic (0.3%) (Figure 4B). This percentage was higher in growth conditions where additional 40 mmHg pressure was added (3.2%).

Conditioned media from MC3T3 cells grown in the bioreactor was also capable of increasing the percentage of multinucleated TRAP positive cells (5.8%). This increase in percentage remained the same regardless of the pressure applied (Figure 4C).

The observation that transition from 0 to 40 mmHg induced higher pro-osteoclastogenic potential in MLO-Y4 cells, but did not impact the pro-osteoclastogenic potential in MC3T3 cells, which is likely due to the MLO-Y4 cells having the osteocyte’s ability to respond to mechanical forces in a superior fashion compared to the MC3T3 cells which are osteoblast-like. This is also reflected in the marked increase (approximately 3-fold) of RANKL production induced by 40 mmHg in the MLO-Y4 cells compared to a limited induction of RANKL in the MC3T3 cells (Figures 3A,C).

## Summary

In summary, we present a working tunable bioreactor that allowed the 3D culture of bone cells embedded in a matrix comprised HA/TCP bone-like materials. This bioreactor was capable of modulating hydrostatic pressure in the presence of FSS. The process to fabricate this bioreactor described, herein, is straightforward and can be achieved by most biological laboratories looking to fabricate their own bioreactor. We were able to demonstrate the utility of this bioreactor in by applying different mechanical forces and two different cell lines that elicited different mechanotransducive responses that promoted osteocyte activity.

The bioreactor reported in this study allowed us to apply a range of physiological pressures from 20 to 60 mmHg above atmospheric pressure. This facilitated further study on how small changes in pressure may affect bone development. This may also be of use in the studying diseases such as prostate cancer where it has been shown that an increase of 40 mmHg in intraosseous pressure induced by tumors inoculated into mouse tibiae was sufficient to increase the expression of pro-metastatic cytokine CCL5 (Sottnik et al., 2015). The FSS cells experienced in the bioreactor was calculated to be approximately 1.16 dynes/cm^2^, while this is lower than what is estimated to be physiological (8–30 dynes/cm^2^) (Weinbaum et al., 1994), and this is within the range of FSS other groups have used (McCoy and O'Brien, 2010).

Cellular response was measured by changes to Runx2, Rankl, Dmp1, Alpl, and Sost expression in response to 3D culture in HA/TCP beads, FSS, and pressure. MC3T3 cells exhibited a phenotypic shift toward a more osteocyte-like phenotype without requiring the application of additional pressure. This is indicated by the downregulation of osteoblast transcription factor Runx2 and osteoblast marker Alpl and the upregulation of pro-osteoclastogenesis marker Rankl and osteocyte markers Dmp1 and Sost (Toyosawa et al., 2001; Jensen et al., 2010; Nakashima et al., 2011; Rutkovskiy et al., 2016; Weivoda et al., 2017). The application of different pressure conditions only altered the Rankl protein level. This suggests that for MC3T3 cells, 3D culture in the presence of HA/TCP beads and FSS was sufficient to induce a phenotypic shift. This observation agrees with previous findings that osteoblasts are more responsive to FSS 20. On the other hand, MLO-Y4 cells appeared to respond to 3D culture, FSS, and changes in pressure. MLO-Y4 3D culture with HA/TCP and FSS was sufficient to downregulate Runx2 and upregulated Dmp1 and Sost but insufficient to induce a change in Rankl. Changes in Sost were observed only at transcript levels, and protein levels remained undetectable. Rankl expression increased at 20 mmHg and did not alter with higher pressure conditions while Dmp1 and Sost expression in MLO-Y4 cells was higher at 20 mmHg pressure and dropped at 40 and 60 mmHg. This suggests that the amount of applied pressure plays an important role in bone homeostasis.

A limitation of this study includes that the spheres were not regularly packed and in some instances cells filled some of these voids (as seen in Figure 2C). This suggests that there could be localized areas of hypoxia. In future studies, staining for hypoxia-inducible factor 1α (HIF-1α) could be used to identify potential hypoxic areas.

The differential responses that MLO-Y4 and MC3T3 exhibit in the presence of FSS and pressure are expected to give the innate differences in the mechanical properties of the environment these cells typically reside at. Osteoblasts are typically encased in a hard bone matrix while osteocytes are embedded within a bone matrix interconnected by a canicular network and are exposed to compressive forces and FSS. Osteoblasts and osteocytes have been shown to respond differently to forces such as FSS or mechanical strain (Owan et al., 1997; McGarry et al., 2005). Our results validate the utility of the bioreactor in conducting future in-depth mechanotransduction studies to further understand the underlying mechanisms behind these differences.

## Conclusion

There is a need for physiological yet manageable *in vitro* bone models to enable in-depth mechanistic studies underlying normal bone development and disease conditions affecting the bone; while several models exist, they are primarily either 2D cell culture plates in the absence of a bone mineral matrix or for those that are in 3D, and they neither recapitulate the structure of bone nor allow for rapid and facile modulation of pressure. In the article, we have described the procedure to fabricate a functioning bioreactor using materials that can be found in most biological laboratories. An advantage of this bioreactor compared to previous models is that it facilitates the culture of osteocytes in a 3D scaffold made of the same substance as bone mineral (i.e., hydroxyapatite) under the supply of constant FSS and pressure, which can be readily altered. We further validated the utility of this bioreactor in studying how an increase in pressure may affect bone remodeling processes. Thus, we have developed a bioreactor that can recapitulate the aspects of osteocyte bone biology.

## Data Availability

The raw data supporting the conclusion of this article will be made available by the authors, without undue reservation.
