# Supplementary material for: A Bioreactor for 3D In Vitro Modeling of the Mechanical Stimulation of Osteocytes
Source: Front Bioeng Biotechnol. 2022 Mar 25;10:797542. doi: 10.3389/fbioe.2022.797542 (PMC8990130; doi:10.3389/fbioe.2022.797542)
Supplement: Supplementary file 1 [file DataSheet1.PDF]

Supplemental Information

A bioreactor for 3D in vitro modeling of the mechanical stimulation of osteocytes

Koh Meng Aw Yong[1,] Eric Horst[2], Dylan Neale[3], Sonya Royzenblat[4], Joerg Lahann[3],[4]
Colin Greineder[5], Megan Weivoda[4,6], Geeta Mehta[2], Evan T. Keller[1,4,*]

[1] Department of Urology, Michigan Medicine, University of Michigan
[2] Department of Material Science & Engineering, University of Michigan
[3] Department of Chemical Engineering, University of Michigan
[4] Biosciences Institute, University of Michigan
[5] Department of Emergency Medicine, University of Michigan
[6] Department of Periodontics and Oral Medicine, University of Michigan
* Correspondence: etkeller@med.umich.edu

Supplemental Table 1.

	Pressure		
	0 mmHg	20 mmHg	40mmHg
β-actin (C_t)	17.26±0.56	16.79±0.14	16.31±0.07
GAPDH (C_t)	18.17±0.73	17.58±0.02	17.26±0.09

Supplemental Table 1. Impact of pressure on housekeeping genes in MLO-Y4 cells. To evaluate for changes in GAPDH and β-actin mRNA expression in MLO-Y4 cells exposed to pressure, we plated $1x10^5$ cells were plated into 2 ml of complete media in 6-well cell culture plates. After 24 hours of incubation, the cells were subjected to constant 0, 20 mmHg or 40 mmHg for 72 hours using the FX-5000C Compression System (Flexcell International Corporation, Burlington, NC, USA), at which time total RNA was collected and 1 µg of total RNA was used to create cDNA and subjected to PCR for GAPDH and β-actin mRNA. Samples were run in triplicate. Data are reported of as mean±SD threshold cycle (C_t).

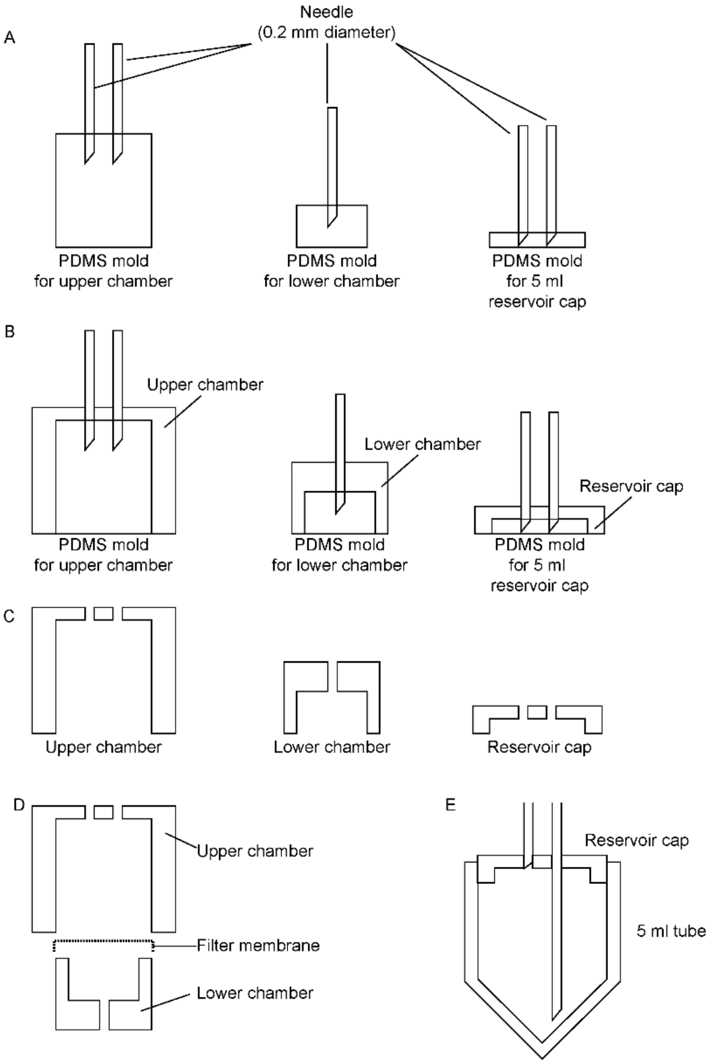

Supplementary Figure 1. Overview of the bioreactor and reservoir fabrication process. A. The molds for the upper chamber (left), lower chamber (middle) and reservoir cap (right) are made using PDMS with needles of 0.2 mm thickness running vertical to the flat surface of each mold. B. How the upper chamber (left), lower chamber (middle) and reservoir cap (right) would appear with the mold. C. After removing molds, the upper chamber will have an open cavity with two channels running vertical in the top wall. The lower chamber will have an open cavity with on channel and the reservoir cap will have an open cavity with two channels. D. Assembly of the bioreactor. The upper and lower chambers are oriented as shown and a filter membrane placed between the

two chambers. By pushing the lower and upper chamber together, the filter is held in place separating the two chambers. E. Assembly of the reservoir. Before placing the reservoir cap in the 5 ml tube as shown, two PTFE tubings are placed in the reservoir cap as shown with one extending to the bottom of the 5 ml tube while the other tubing is placed into the cap not extending past the opening.

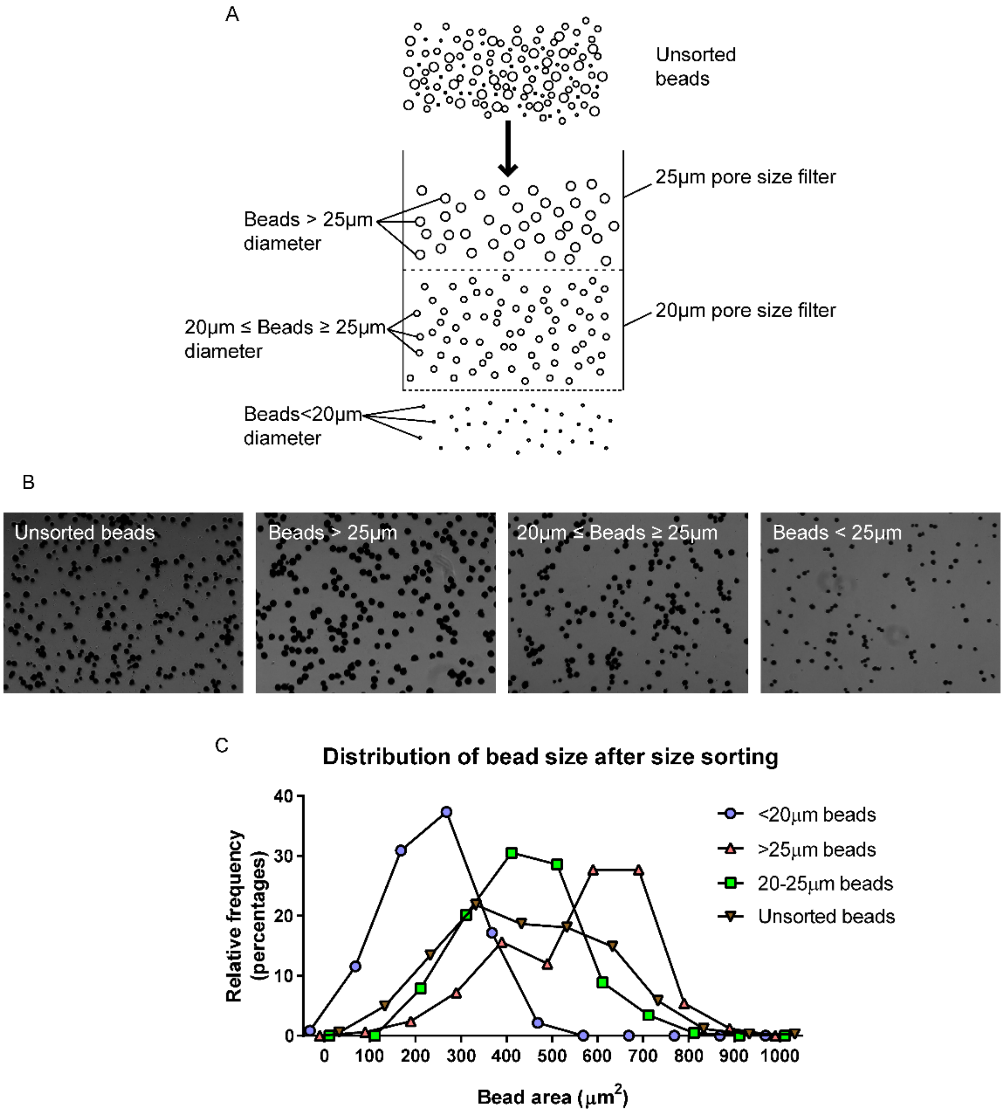

Supplementary Figure 2. Size sorting of HA/TCP beads. A. Unsorted beads are first passed through a 25 µm pore size filter. The flow-through is next passed through a 20 µm pore size filter. The beads retained on the 20 µm pore size filter are harvested and resuspended in a 75 % ethanol solution for sterilization. B. Phase images of the unsorted beads before size filtration (left); the beads retained on the 25 µm pore size filter (second from left); beads retained on the 20 µm pore size filter (third from left) and the flow through from the 20 µm pore size filter (right). C. Distribution graph of the bead diameters from each fraction of the filtrate.

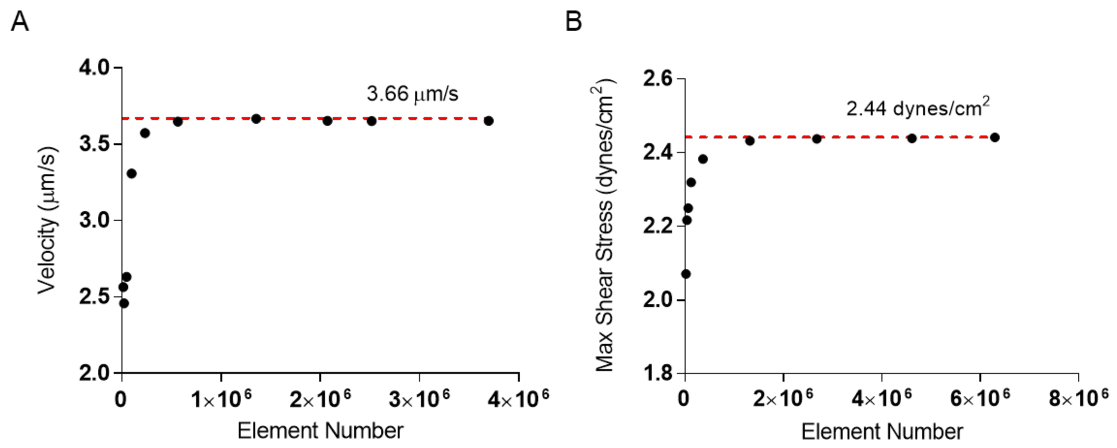

Supplementary Figure 3. Simulation Mesh Analysis. A. Mesh analysis of fluid velocity sampled before the HA layer. B. Mesh analysis for maximum surface shear stress on the model osteocyte.

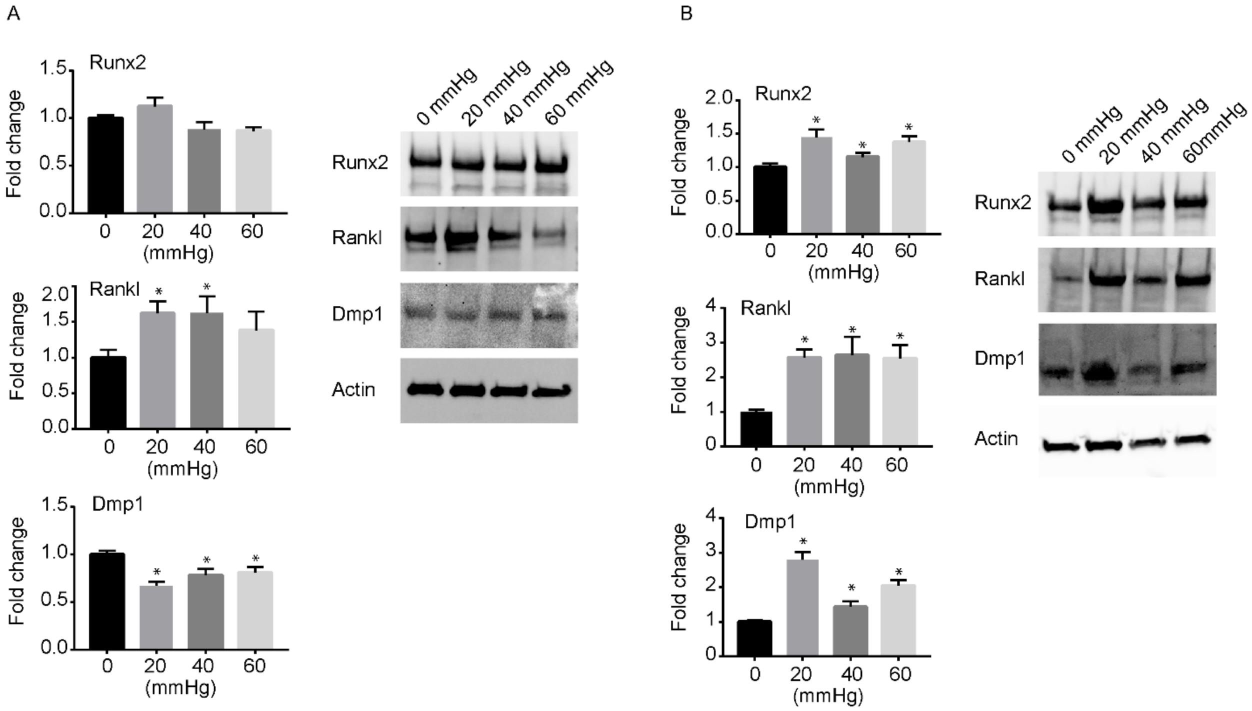

Supplementary Figure 4. Expression of bone remodeling genes Dmp1, Rankl and Runx2 in response to changes in pressure. A. Transcript levels of Runx2 (top left), Rankl (middle left) and Dmp1 (bottom left) in MC3T3 cells grown in bioreactor and pressure conditions of 0, 20, 40 and 60 mmHg. Protein levels of the same genes are shown in the immunoblot on the right. B. Transcript levels of Runx2 (top left), Rankl (middle left) and Dmp1 (bottom left) in MLO-Y4 cells grown in bioreactor and pressure conditions of 0, 20, 40 and 60 mmHg. Protein levels of the same genes are shown in the immunoblot on the right. Data are presented as mean ± SD from three independent experiments. *$p \leq 0.05$ vs. 0 mmHg.
